# Real‐time tracking of fibrinolysis under constant wall shear and various pulsatile flows in an in‐vitro thrombolysis model

**DOI:** 10.1002/btm2.10511

**Published:** 2023-04-11

**Authors:** Ziqian Zeng, Alexei Christodoulides, Nathan J. Alves

**Affiliations:** ^1^ Department of Emergency Medicine Indiana University School of Medicine Indianapolis Indiana USA; ^2^ Weldon School of Biomedical Engineering Purdue University West Lafayette Indiana USA

**Keywords:** drug delivery, fibrinolysis, in‐vitro model, physiological model, shear flow, thrombolysis

## Abstract

A great need exists for the development of a more representative in‐vitro model to efficiently screen novel thrombolytic therapies. We herein report the design, validation, and characterization of a highly reproducible, physiological scale, flowing clot lysis platform with real‐time fibrinolysis monitoring to screen thrombolytic drugs utilizing a fluorescein isothiocyanate (FITC)‐labeled clot analog. Using this Real‐Time Fluorometric Flowing Fibrinolysis assay (RT‐FluFF assay), a tPa‐dependent degree of thrombolysis was observed both via clot mass loss as well as fluorometrically monitored release of FITC‐labeled fibrin degradation products. Percent clot mass loss ranged from 33.6% to 85.9% with fluorescence release rates of 0.53 to 1.17 RFU/min in 40 and 1000 ng/mL tPa conditions, respectively. The platform is easily adapted to produce pulsatile flows. Hemodynamics of human main pulmonary artery were mimicked through matching dimensionless flow parameters calculated using clinical data. Increasing pressure amplitude range (4–40 mmHg) results in a 20% increase of fibrinolysis at 1000 ng/mL tPA. Increasing shear flow rate (205–913 s^−1^) significantly increases fibrinolysis and mechanical digestion. These findings suggest pulsatile level affects thrombolytic drug activities and the proposed in‐vitro clot model offers a versatile testing platform for thrombolytic drug screening.

## 1 INTRODUCTION

**FIGURE 1:**
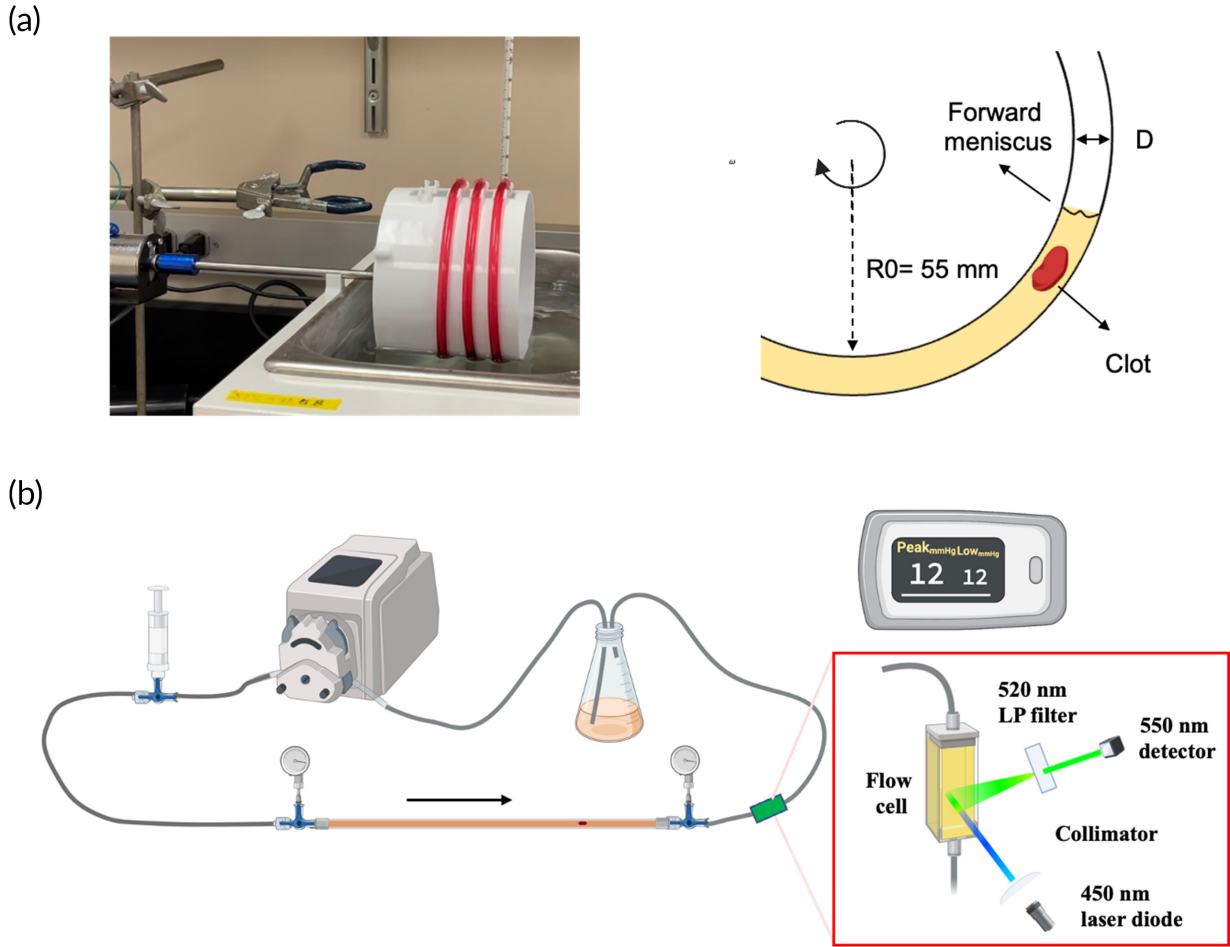
Schematic representation of (a) Chandler loop and (b) Real‐Time Fluorometric Flowing Fibrinolysis Assay. Components from left to right are flow dampener, peristaltic pump, pressure sensor A (before clot), reservoir, pressure sensor B (after clot), and fluorometer.

Thrombosis is the pathologic formation of a blood clot that obstructs flow through vasculature. Complications of thrombosis can be life‐threatening, such as ischemic stroke, myocardial infarction, and pulmonary embolism (PE). The mainstay of thrombosis treatment is either through mechanical thrombectomy, which requires a specialized device and an experienced clot retriever‐operator, or by intravenous infusion of thrombolytic agents.^1^, ^2^ Both of the aforementioned approaches maintain inherent risks, bleeding being the primary.^3^ Numerous in‐vitro drug testing methods have been developed to assist in the screening and development of novel thrombolytic agents. A static in‐vitro clot lysis assay using a statically formed clot is the most common protocol found in the literature.^4^, ^5^ Tests are performed by adding drugs to preformed clots and tracking changes in clot weight to depict drug efficacy.^5^ This method can address scenarios where diffusion is the dominant biophysics of thrombolysis, for example, in patient with deep vein thrombosis (DVT) but is limited to address patients with PE or stroke not to mention arterial thrombosis. Nevertheless, most DVT patients are not recommended for thrombolytic therapy, according to the guideline by the American Society of Hematology.^6^ Static thrombolysis inherently ignores human hemodynamics where parameters like trans‐thrombus pressure drop, and turbulent flow can dramatically affect clot permeation, resulting in distinctive drug efficacy profiles.^7^, ^8^, ^9^ Properties of statically formed clots are different from those of native thrombi due to the lack of shear effects during clotting. Flowing blood even at low shear conditions (<500 s^−1^) promotes the formation of distinct clot motifs rather than complete stasis.^9^, ^10^, ^11^ Thus, devices have been engineered in attempts to achieve more relevant physiological digestion conditions.^7^ Microfluidic‐related assays are useful in studying thrombosis progression, given their highly ordered flow patterns and ease of imaging. Clots formed in these devices tend to be dissimilar to native thrombi due to the nature of device lumen size and difference in flow dynamics.^12^, ^13^ Utilizing a Chandler loop device to form clot analogs and study clot digestion has gained popularity in thrombosis societies in recent years (Figure 1a).^14^, ^15^ Clot analogs formed in the Chandler loop have revealed a good resemblance to native venous thrombi, arterial thrombi, and pulmonary emboli.^10^ The Chandler loop also allows for clot digestion under shear; however, its over‐simplified nature makes it a less representative model since the setup lacks important physiological circulatory components such as a reservoir, pressurized flow conditions.

Importantly, while studying steady‐state shear or continuous pressure effects on clot digestion is useful, modeling flow pulsatility generated by the human heart also demands research attention as it more closely represents in‐vivo hemodynamics. Although pulsatile flow and cyclic wall stress have long been considered to impact in‐vivo drug or enzyme activities, only a few studies have explored this relation in the past and the information on how pulsatile pressure amplitude (the pulsatile pressure difference between systolic and diastolic pressure over a cardiac cycle) affects the thrombolytic efficacy is missing.^16^, ^17^, ^18^

Given the drawbacks and benefits of existing methods, developing a drug screening platform that combines a physiologically relevant clot and biomimetic flow is essential to more meaningful drug screening. This model may not eliminate the need for preclinical animal testing but can exclude inefficient agents earlier in the drug development pipeline to expedite the drug evaluation process. This study reports the design, validation, and characterization of an in‐vitro thrombolysis model for thrombolytic drug screening. This model is a tubing‐based system that incorporates a peristaltic pump, a flow dampener, a temperature‐controlled reservoir, pressure sensors, a fluorometer, and a Chandler loop‐formed clot analog (Figure 1b). The system can be used to develop a laminar flow at a constant shear or offer more human‐relevant pulsatile flows through engineering scaling. According to the Buckingham‐Pi theorem, the kinematic similarity of a flow can potentially be achieved by having identical dimensionless factors. These include the ratio of length scales to ensure a dimensional similarity, Reynolds number to have a similar flow pattern, and Fanning friction factor to have a comparable fluid shear effects on lumen surfaces. When the pulsatile flow is relevant, Womersley number should also be matched to that of the target in‐vivo condition to keep dynamic similarity.^19^, ^20^ In addition, the clot analog utilized is fluorescently tagged by using a previously reported low‐impact labeling strategy to facilitate real‐time tracking of fibrinolysis.^21^ The entire system will be referred to as Real‐Time Fluorometric Flowing Fibrinolysis assay (RT‐FluFF assay) for the purposes of this paper. A series of characterization experiments were conducted to validate flow dynamics, impact‐free fluorescent clot labeling, and real‐time fluorescence tracking underflow. Digestion experiments were first conducted at a constant shear to demonstrate the capability of differentiating increasing levels of thrombolytic agents on clot digestion efficacy. To extend the capability for offering clinically relevant pulsatile flows, we tuned the system to mimic human MPA to study pulsatility impact on thrombolysis as thromboemboli lodged near the main pulmonary artery (MPA) account for most of the PE‐related mortality.^22^, ^23^

## 2 RESULTS

### 2.1 Generating a physiologically relevant clot analog

A common means of introducing fluorescence into clot analogs for clot lysis study is using fluorescein isothiocyanate‐labeled‐fibrinogen (FITC‐Fg).^21^, ^24^, ^25^ Concentrations of FITC‐Fg could range anywhere from 0.0075 to 0.6 mg/mL using 1–15 FITC per fibrinogen with no justifications as to why a particular concentration or labeling density is preferred.^9^, ^26^, ^27^, ^28^ To create a truly representative thrombolysis system relying on FITC‐Fg labeling, we had previously demonstrated endogenous Fg to FITC‐Fg = 50:1 present minimal impact and 10:1 present small deviation on cell‐free fibrin clot properties.^21^ Herein, we expanded to optimize the ratio of endogenous Fg to FITC‐Fg present in whole blood samples as the introduction of modified Fg could largely affect clot structure. We aimed for the highest level of fluorescence labeling while maintaining clot integrity and architecture. Clotting mixtures were prepared by adding FITC‐Fg (14‐FITC per fibrinogen) to whole blood at ratios of native Fg to FITC‐Fg at 1:0 (control), 50:1, 10:1, and 5:1. Thromboelastography (TEG) was elected to compare clotting parameters across groups. Increasing the amount of FITC‐Fg added to whole blood led to decreased maximum amplitude (MA), increased time to maximum amplitude (TMA), and decreased angle (Figure S1). At native Fg to FITC‐Fg = 10:1, samples showed minimally reduced MA (5.6%, *p* = 0.0208), similar TMA and angle values compared to the control group (Figure S1).

**FIGURE 2:**
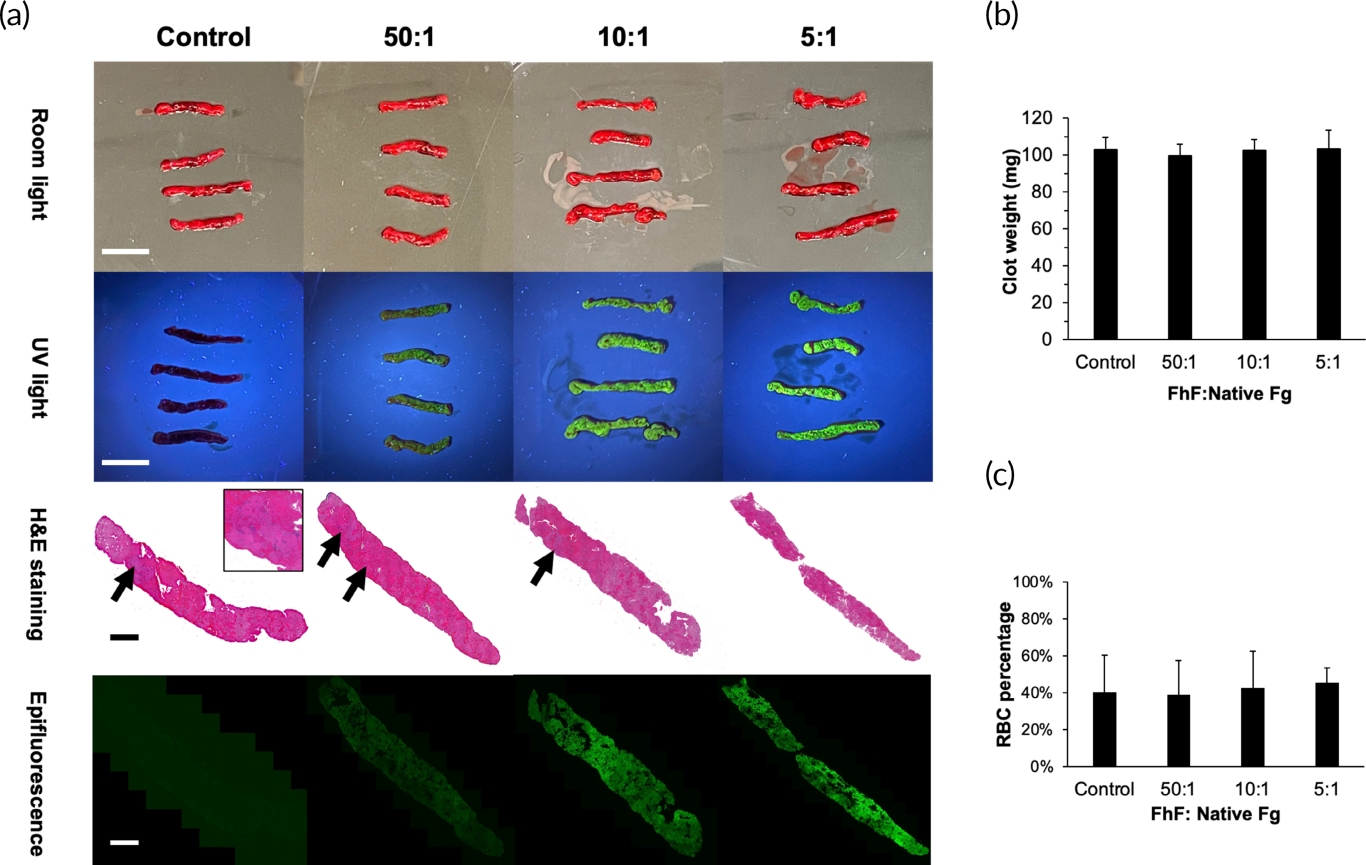
Whole blood clot analog generation and characterization. (a) Gross images of clots formed in the Chandler loop using varying FhF ratios under room light (top row) versus UV light (second row). Scale bars represent 20 mm. H&E (third row) and epifluorescence images (fourth row) acquired of Chandler loop clots at the same view. Scale bars represent 2 mm. For H&E staining, RBCs, fibrins, and WBCs were stained in dark red, pink, and blue. Black arrow indicates packed fibrin motifs. Note the loss of packed fibrin motifs in the 5:1 group, indicating a significant perturbation of clot architecture. (b) Masses and (c) RBC percentage of clots formed in the Chandler loop utilizing respective ratios of FITC‐Fg. No significant differences (*p* > 0.05) were found for clot mass and RBC percentage across groups. H&E, hematoxylin and eosin; RBC, red blood cell; WBC, white blood cell.

The proposed thrombolysis system utilizes clot analogs formed under pulmonary shear (464 s^−1^). Thus, a Chandler loop device was utilized to further characterize labeled clots at the mentioned FITC‐Fg ratios. The final clot masses did not deviate significantly across groups; the 5:1 group (103.6 mg) and the 10:1 group (102.6 mg) had nearly identical masses to controls (Figure 2b; 103.2 mg). Subsequent digestion of these clots was further performed under shear in the Chandler loop with Alteplase (tPa). Clot mass loss after digestion was not significantly different (Figure S2). Grossly, all clots showed similar appearance, with exposure to UV light highlighting increasing fluorescence intensity expected from increasing FITC‐Fg incorporation at higher ratios (Figure 2a). Noticeably, clot histology by hematoxylin and eosin (H&E) staining revealed the loss of packed fibrin motifs at the 5:1 ratio group, regions that were seen in all other groups (Figure 2a).

Quantitative analysis of clot components was conducted using H&E images through ImageJ. Five regions of interest (ROIs, 2 mm × 2 mm) were selected at random and cropped from the H&E images for clot structural assessment. No significant difference was found across groups on clot red blood cell (RBC) percentage (*p* = 0.67) (Figure 2c). Clot homogeneity was further assessed using coefficient of variance (COV) calculated by dividing the standard deviation by the average RBC percentages of the selected five ROIs for each clot. The 5:1 ratio group showed the smallest values (COV = 0.179) indicating a much more homogenous clot structure compared to clots formed at 10:1 (COV = 0.468), 50:1 (COV = 0.474), and control (COV = 0.491) groups. Epifluorescence conducted on microtomes of the same clots from H&E staining displayed uniform FITC‐Fg incorporation throughout the clots (Figure 2a). Both the 10:1 and 5:1 groups appeared to have adequate levels of fluorescence, while the 50:1 group showed a huge reduction in fluorescence intensity which was calculated to give a low signal‐to‐noise ratio for the developed RT‐FluFF system. In all, the 10:1 ratio of FITC‐Fg was utilized for all subsequent testing as this ratio best balanced increased fluorescence incorporation with minimal perturbation of clotting/clot parameters.

### 2.2 RT‐FluFF assay device characterization

**FIGURE 3:**
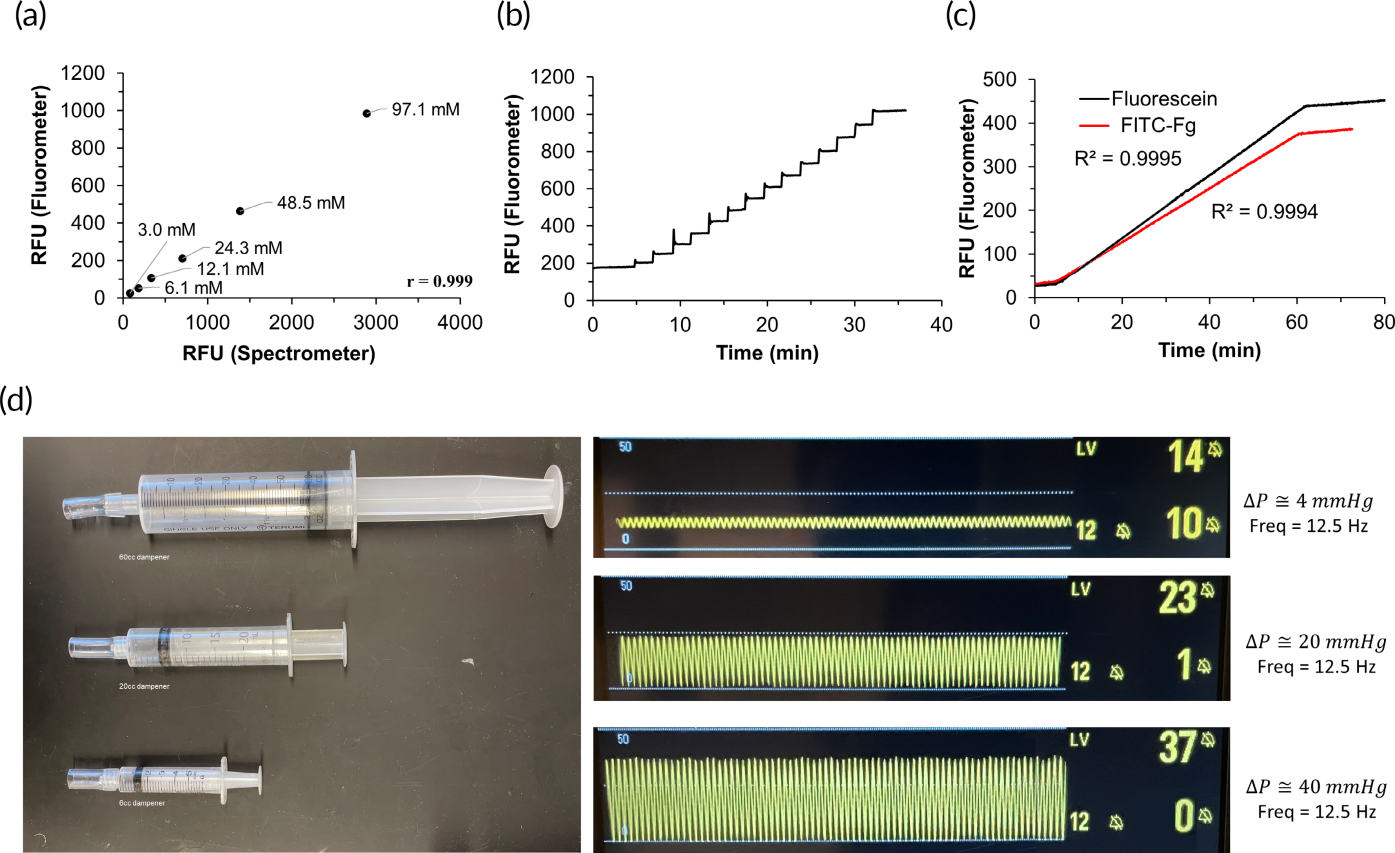
Device characterization. (a) Comparison between spectrophotometer and fluorometer in quantifying fluorescence of a serial dilution of FITC‐Fg. (b) Stepwise injection of fluorescein into flowing plasma with fluorometer data acquisition. (c) Continuous infusion of either FITC‐Fg or fluorescein into plasma with fluorometer data acquisition. (d) Pressure waveforms (by pressure sensor B) reveal various pulsatile pressure amplitudes (4, 20, and 40 mmHg) via the implementation of different‐sized flow dampeners (60, 20, and 6 cc, correspondingly). FITC‐Fg, fluorescein isothiocyanate‐fibrinogen.

The RT‐FluFF assay utilizes a system that incorporates a peristaltic pump with an adjustable pulsatile‐flow dampener that can produce physiologic levels of shear or flow dynamics in a versatile system allowing for great interchangeability of tubing lumen size and geometry (Figure 5). Pressure sensors were placed before and after the clot location to quantify flow dynamics. Finally, an in‐line fluorometer was incorporated to allow for real‐time monitoring of fluorescence in the fluid flow‐by. The developed fluorometer was validated via both static and flowing samples. A correlation test was performed against a SpectraMax‐M5 spectrophotometer using serial diluted FITC‐Fg at static conditions where *r* = 0.999 was reported. The in‐line fluorometer captured a 62.8 ± 7.4 increase (COV = 11.8%) after stepwise injections of 5 μL fluorescein (4.2 mmol) in the flowing system. Equivalent total fluorescein molecular amounts (0.021 μmol) of fluorescein and FITC‐Fg were further compared in the system at an infusion rate of 1000 μL/h. Similar RFU increase rates were noticed, with 7.16 RFU/minute for fluorescein and 6.04 RFU/minute for FITC‐Fg (Figure 3c). Calculation of fluorometer RFU contribution from FITC‐Fg introduction showed that 1 RFU corresponded to a rise in system FITC‐Fg concentration by approximately 0.1 nM.

The RT‐Fluff system can be adapted to provide for both laminar and pulsatile flow dynamics through the utilization of a pulsatile‐flow dampener. To achieve laminar flow conditions, we built a dampener using a 60‐cc syringe. This dampener largely reduces pressure fluctuations from peristalsis to establish an approximately laminar flow in the region of the system where clots were loaded. Laminar flow dynamics were confirmed by matching monitored pressure drops across the two sensors to a theoretical value calculated through the Hagen–Poiseuille equation. The dampening effect was further demonstrated by having up to a <4 mmHg difference between systolic/diastolic pressures at any of the two sensors, compared to a ~55 mmHg average fluctuation in un‐dampened conditions within ~300–1000 s^−1^ shear (Figure S3). Importantly, the developed system was able to withstand pressures of up to ~140 mmHg at extremes; however, running pressures were generally kept between 8 and 20 mmHg to simulate physiological pressures expected in human pulmonary arteries (Figures S4 and S5). One major benefit of the RT‐FluFF system is it can provide for amplitude‐adjustable pulsatile flows. An MPA‐relevant pulsatile flow was established through matching dimensionless factors via selected model components and a peristaltic pump rate of 187 RPM to give an average wall shear rate of 913 s^−1^. Flow parameters used for factor calculations were collected from normal patient MPA data (Table S1). Three dampeners (60, 20, and 6 cc) were utilized to create three cyclic pressure amplitudes at 913 s^−1^ corresponding to 4, 20, and 40 mmHg, respectively (Figure 3d).

Final system characterization revolved around understanding how a clot would behave under various conditions of shear before the addition of thrombolytic drugs. Chandler loop‐formed clots were formed with either no FITC‐Fg, 5:1, or 50:1 ratio of FITC‐Fg, that is, the upper and lower limits previously described. Analysis of the percent change in length between the groups at various rates of shear, 0–900 s^−1^, showed that clots underwent highly reproducible degrees of elongation with respect to a given shear rate (Figure S6). In other words, clot behavior was very consistent between varying levels of shear, ensuring that clots would be exposed to similar mechanical forces if thrombolytic drugs were introduced.

### 2.3 Quantifying thrombolysis of whole blood clots in real‐time under constant wall shear flows

**FIGURE 4:**
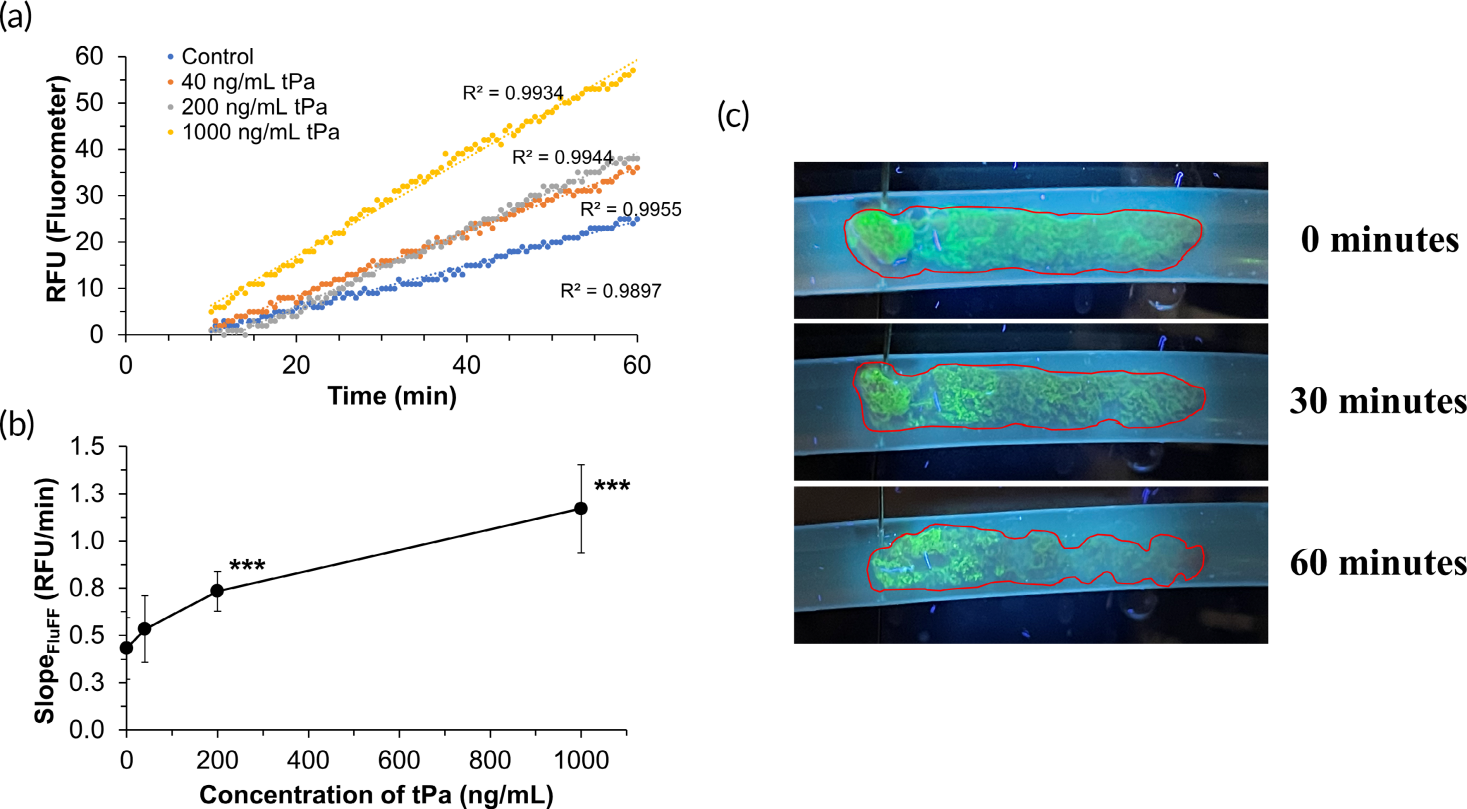
(a) Representative scatter plot outlining rise in fluorescence over the course of thrombolysis in respective tPa conditions in the RT‐FluFF assay at a constant shear rate of 398 s^−1^. (b) Plot of linear‐phase slopes captured in RT‐FluFF assay as dictated by tPa concentration (0, 40, 200, and 1000 ng/mL). (c) Representative example of gross changes that clots undergo during digestion. Pictured is the 200 ng/mL tPa condition illuminated under UV light. For data presented here, a single asterisk denotes a *p* value < 0.05, double asterisk denotes a *p* value < 0.01, and triple asterisk signifies a *p* value < 0.001. RT‐FluFF, Real‐Time Fluorometric Flowing Fibrinolysis.

Noting the consistency of both the in‐line fluorometer as well as FITC‐Fg‐labeled clot analogs, we first proceeded to introduce our clot analogs into the RT‐FluFF assay for thrombolysis at a constant shear level (398 s^−1^). The clot analogs were fixed at a 36‐cm entrance length away from the first pressure sensor (sensor A) to ensure fully developed laminar shear. Clot digestion experiments were conducted using plasma and varying tPa concentrations: 0, 40, 200, and 1000 ng/mL. Thrombolysis was monitored by a rise in fluorescence within the plasma as FITC‐labeled fibrin degradation products were released from the clots themselves, and rates of digestion could be extrapolated from the slopes of the linear phases of the generated scatter plots (Figures 4a and S7). Of note, the linear phases generally developed 10 min from the time tPa was added and flow was stabilized. In addition to drastic rises in fluorescence, thrombolysis was also readily observable as fibrin degradation led to changes in both clot surface morphology and structural stability in the face of mechanical shear forces (Figure 4b). Increasing concentrations of tPa led to consistently noticeable rises in rate of clot digestion—that is, greater rates of FITC‐Fg release (Figure 4c). The rate of digestion in the 40 ng/mL tPa (0.53 RFU/min) group did not significantly differ (*p* value = 0.308) from the background rise in fluorescence measured from plasma in the control group (0.43 RFU/min). Addition of 200 ng/mL of tPa led to a statistically significant (*p* value = 0.008) rise in the rate of thrombolysis up to 0.73 RFU/min with 1000 ng/mL of tPa further raising the slope to 1.17 RFU/minute (*p* value < 0.001).

**FIGURE 5:**
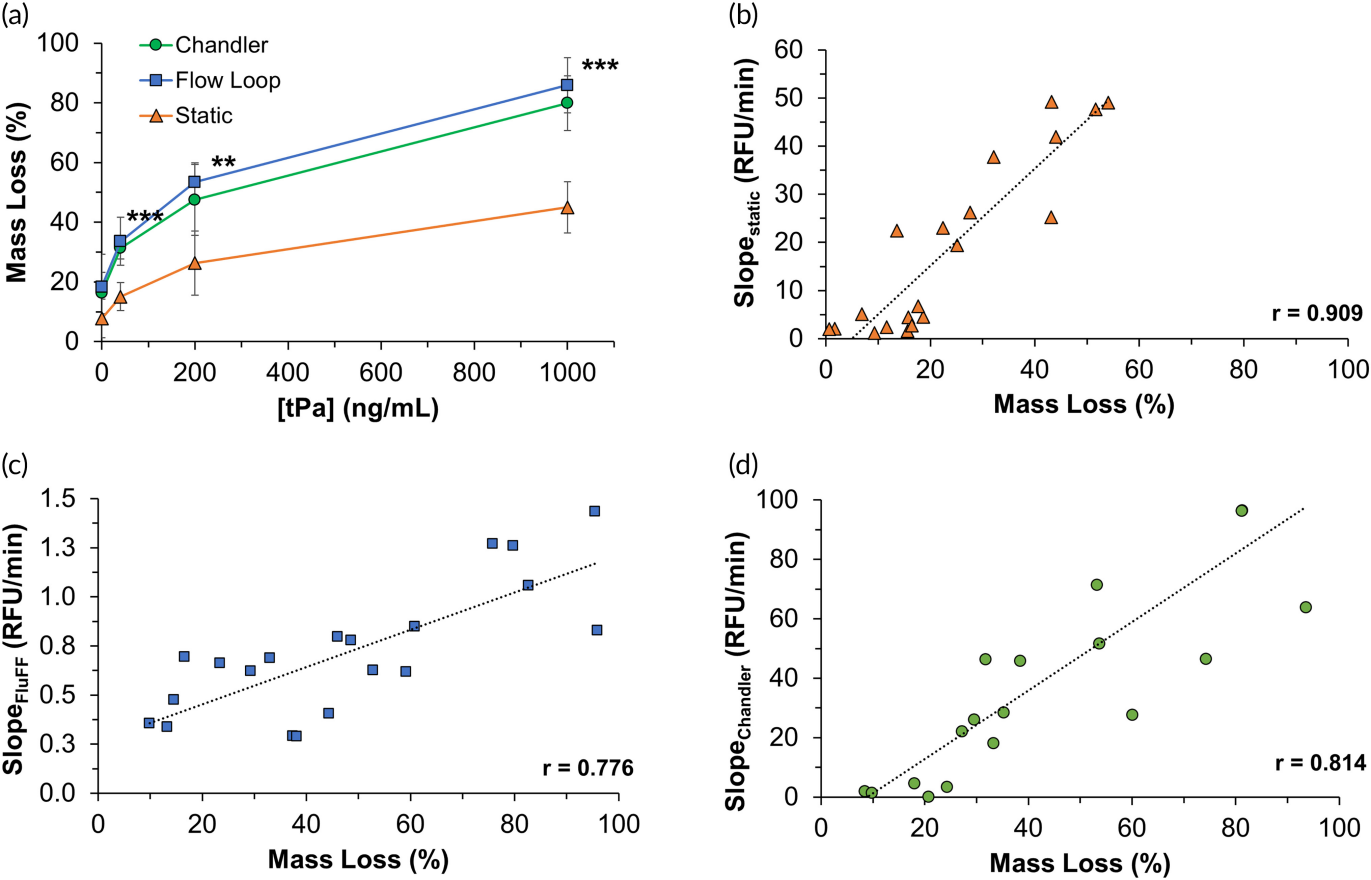
Quantifying degree of clot lysis and correlation to rises in fluorescence. (a) Percent clot mass lost based on various concentrations of tPa (0, 40, 200, and 1000 ng/mL) and varying thrombolysis modalities (Chandler loop at 464 s^−1^, RT‐FluFF assay at 398 s^−1^, and static conditions). (b) Correlation analysis for static digestion. RFU as tracked by the spectrophotometer. (c) Correlation analysis for the RT‐FluFF assay. RFU as tracked by the fluorometer. (d) Correlation analysis for Chandler loop digestion. RFU as tracked by the spectrophotometer. For data presented here, a single asterisk denotes a *p* value < 0.05, double asterisk denotes a *p* value < 0.01, and triple asterisk signifies a *p* value < 0.001. RT‐FluFF, Real‐Time Fluorometric Flowing Fibrinolysis.

We then investigated digestion differences associated with pressure‐driven flow in the RT‐FluFF versus non‐pressure‐driven flow in the Chandler loop and no flow under static conditions. Similar to the data seen in the pressure‐driven flow of RT‐FluFF, the rate of FITC‐Fg release increased with increasing tPa concentrations for both static and Chandler loop conditions (Figure S8). Once again, rises in the rates of FITC‐Fg release start at the 40 ng/mL tPa condition for both static and Chandler loop digestion assays. These differences were both statistically significant compared to their respective control groups. The 200 and 1000 ng/mL tPa conditions also reached statistical significance in the static digestion and Chandler loop digestion assays (Figure S8). In addition to collecting rates of FITC‐Fg rise as a proxy for thrombolysis, degree of clot digestion was also assessed based on the percentage of mass lost during digestion. Interestingly, across all the tPa concentrations, digestion in the RT‐FluFF assay was consistently slightly higher than in the Chandler loop; however, these differences were minor and statistically not different (Figure 5a). For example, percent clot mass lost at the 1000 ng/mL tPa condition was 79.9% in the Chandler loop versus 85.9% in the RT‐FluFF assay (*p* value = 0.564). Large reductions in percent clot mass loss were seen when comparing either of the dynamic digestion assays with respective static conditions (Figure 5a). Comparing the 200 ng/mL tPa condition across all digestion modalities, we saw a 26.4% reduction in clot mass for the static condition in contrast to 47.5% and 53.5% in the Chandler loop and RT‐FluFF assay, respectively (*p* value = 0.003). Of note, although not statistically significant, a ~10% increase of percent mass was seen in both dynamic conditions compared to the static condition at the control group which was majorly attributed to mechanical digestion.

Correlation analyses were performed between the percent mass lost during digestion and the rates of fluorescence rise from FITC‐Fg release in each respective digestion assay. The strongest correlation was seen in the static digestion assay where a linear correlation coefficient of 0.91 was seen between percent mass loss and the rate of fluorescence rise during thrombolysis (Figure 5b). Both dynamic digestion conditions exhibited similarly strong linear correlations to one another, albeit less than the static condition. The correlation coefficient for the RT‐FluFF assay was 0.78 compared to 0.81 in the Chandler loop (Figure 5c,d).

### 2.4 Quantifying fibrinolysis of plasma clot in real‐time under pulsatile flows

A critical question that remained for developing the in‐vitro thrombolysis assay was whether pulsatile flow affects fibrinolysis. Thus, our study explored the impact of various levels of pulsatility on tPA‐induced fibrinolysis. To avoid confounding contributions by RBCs and platelets, fluorescently labeled platelet‐free plasma clots (native Fg to FITC‐Fg = 10:1) were employed. Three pressure amplitudes (4, 20, 40 mmHg) were compared on clot digestion with or without thrombolytic drugs at an average shear of 913 s^−1^. Additional pump rate setups that are 205 s^−1^, 523 s^−1^, and a 1 Hz on/off 523 s^−1^, were also explored to comprehensively study pulsatile flow impact. Rates of fluorescence release upon digestion were derived from fluorescence tracing curves monitored by the in‐line fluorometer to directly indicate clot digestion by thrombolytic drugs.

**FIGURE 6:**
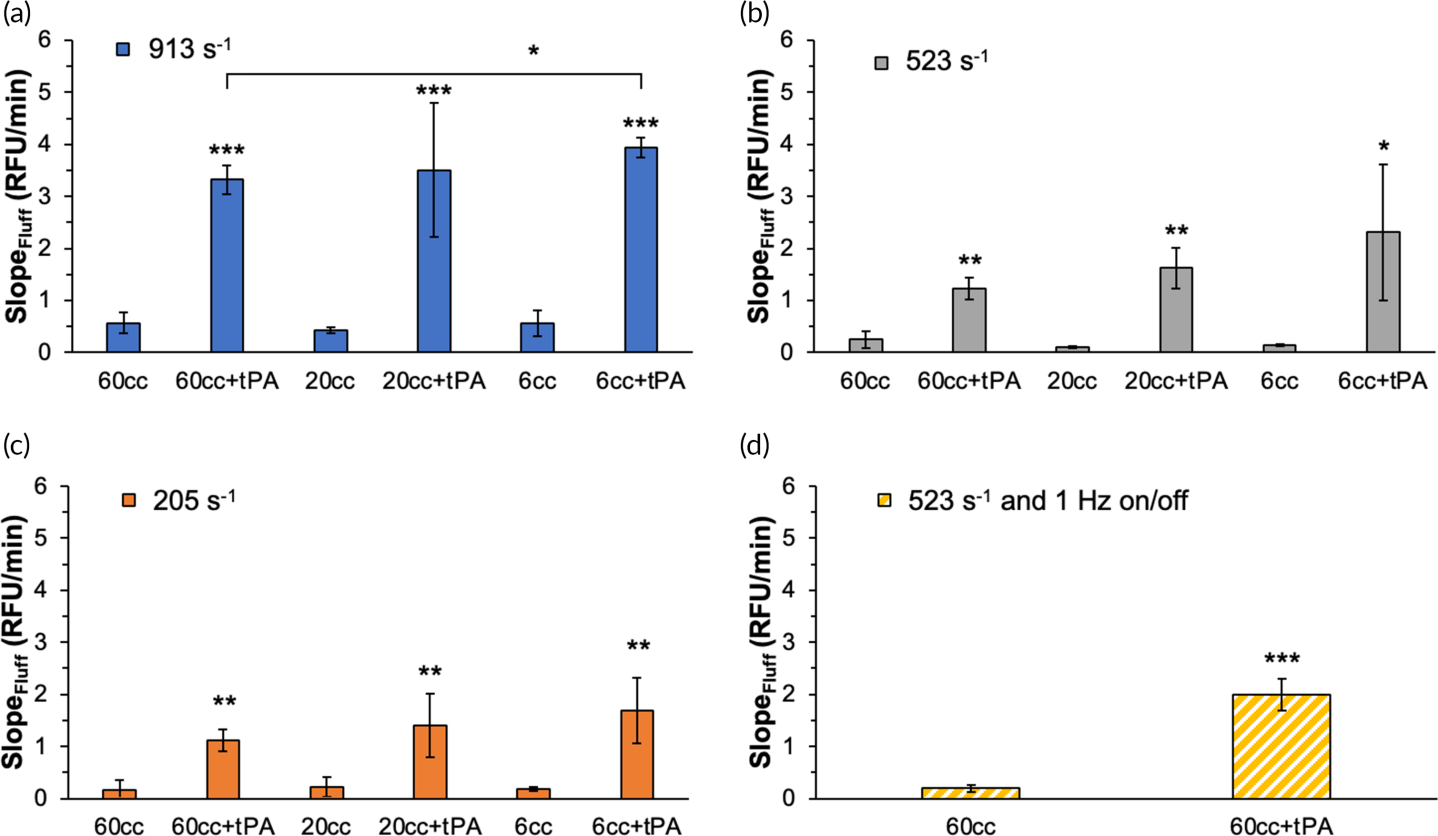
Clot digestion slope (RFU/min) of fluorescently labeled platelet‐free plasma clots in the RT‐FluFF assay were compared across three dampeners settings at the average wall shear = (a) 913 s^−1^ (60 cc, 4 mmHg; 20 cc, 20 mmHg; and 6 cc, 40 mmHg) (b) 523 s^−1^, (c) 205 s^−1^, and (d) 523 s^−1^ 1 Hz on/off setup. Samples were either 0 tPA controls or 1000 ng/mL tPA. Significant differences between 0 and 1000 ng/mL tPA were shown. Single asterisk denotes *p* value < 0.05, double asterisk denotes *p* value < 0.01, and triple asterisk denotes *p* value < 0.001. RT‐FluFF, Real‐Time Fluorometric Flowing Fibrinolysis.

Significant differences were observed across control and 1000 ng/mL tPA at all flow conditions (Figure 6). At 913 s^−1^, the MPA relevant flow dynamic condition, an increasing trend of tPA‐induced clot digestion rate (19% increase of Slope_FluFF_, *p* = 0.0013) was observed when pulsatile pressure amplitude increased from 4 to 40 mmHg or dampener size decreased from 60 to 6 cc (Figure 6a). A similar trend was also observed at 523 s^−1^ (89% increase, Figure 6b) and 205 s^−1^ (51% increase, Figure 6c) when comparing clot digestion rates at 60 and 6 cc, although no statistical significance (*p* > 0.05) was noted because of the large variation in these experimental groups. No such trend was shown in zero exogenous tPA‐added control groups at these flow conditions (Figure 6b,c). This indicated that increasing pressure amplitude at a given flow rate might not increase mechanical digestion but could result in an improved thrombolytic drug efficacy. At any given dampener size, clot digestion rates were higher at 913 s^−1^ than at 205 s^−1^ in all groups. Strong statistical significances were observed in tPA groups at 60 cc (*p* = 0.0001), 20 cc (*p* = 0.0107), and 6 cc dampeners (*p* = 0.006), and no tPA control groups at 60 cc (*p* = 0.017) and 6 cc dampeners (*p* = 0.015). This demonstrated that a higher average wall shear stress or flow rate could contribute to increased mechanical digestion and increased drug‐induced thrombolysis. Similar results and trends were observed in Mass loss% and clot digestion% as clot digestion rate results in all experiments (Figure S9).

Even though matching dimensionless factors allows the production of similar flow dynamics, all pulsatile frequencies employed in this study were higher than that of the human heart. To directly compare human‐relevant pulsatility on thrombolysis, an additional experiment was conducted by adding a human heartbeat pulsation rate (1 Hz) to the highly dampened (60 cc dampener) 523 s^−1^ averaged wall shear condition. The pump speed of the heartbeat setup is doubled to give an equivalent volumetric output as the highly dampened 523 s^−1^ and noted as 523 s^−1^ and 1 Hz on/off. tPA‐induced clot digestion rates were 63% higher (*p* = 0.0165) in the heartbeat setup than in the highly dampened 523 s^−1^, while an inversed result (*p* = 0.641) was seen when comparing no exogenous tPA control groups. These results further confirmed that the addition of pulsatility contributes to an improved drug‐induced fibrinolysis.

## 3 DISCUSSION

### 3.1 Optimization of FITC‐Fg labeling

One of the aims of the current study was to characterize and validate the design of a physiologically relevant in‐vitro model for thrombolytic drug screening. Fibrinolysis is an important step in the dissolution of any thrombus and our study elected to track fibrinolysis via fluorescence release for more informative clot digestion tracking. Ideal clot reporters should have a minimal labeling impact on clot properties while maintaining high fluorescence intensity for ease of reporting and imaging. In prior studies, we have reported the effect of varying multiple clotting components, including reporter conjugated fibrinogen, on fibrin clot properties.^21^, ^29^ This study extended the scope and explored the impact of varying FITC‐Fg levels on whole blood clots. Based on our TEG results, Chandler loop clot weights/appearances/fibrinolysis behavior, and clot H&E histology, our proposed optimal labeling method is to mix whole blood with 14 FITC labels per human fibrinogen to a final concentration of 0.3 mg/mL. This added exogenous FITC‐Fg corresponds to one‐tenth of the average 3 mg/mL fibrinogen content expected in pooled whole blood from healthy volunteers.^30^ Based on our results, this 10:1 ratio of native fibrinogen to FITC‐Fg corresponds with minimal perturbation of clotting parameters and clot histology/appearance while maximizing fluorescence intensity. Lower fluorescence intensity is to be expected if lower FITC per fibrinogen molecules or lower overall FITC‐Fg concentrations are used to achieve a similar impact‐free labeling. On the other hand, addition of greater amounts of reporter begin to drastically impact clotting parameters as well as the underlying structure of the clot itself. The above was suggested both through TEG results, particularly the fall in MA, prolongation of TMA, and fall in angle, in addition to H&E analysis of the 5:1 FITC‐Fg group that displayed alterations in fibrin organization. In particular, H&E analysis of the 5:1 FITC‐Fg group demonstrated the impaired formation of fibrin‐rich regions expected within physiologic clots. Lack of such packed fibrin regions impacts the physical properties of the clot, particularly its mechanical strength, as we experienced during the clot handling process.^31^ Interestingly, studies done on the effects of fibrin carbamylation, the reaction that FITC undergoes with fibrinogen, have shown nearly identical results to ours in terms of perturbations in clotting parameters. Binder et al. showed that carbamylation of fibrin led to decreased rates of polymerization, decreased fibrin cross‐linking, and even increased propensity to clot lysis.^32^, ^33^

Our proposition for the ideal ratio of FITC‐Fg to native fibrinogen should not be viewed as the ultimate standard to be utilized across all assay designs considering that various assays might demand varying levels of reporters. Rather, this data should be used as a guide to others if their aim is to generate physiologically relevant clot analogs for subsequent analysis of fibrinolysis. Additionally, one could easily propose using alternative reporters, labeling strategies, or increasing the utilized FITC‐Fg concentration at the expense of decreasing the number of FITC molecules per fibrinogen, a possibility we did not explore in this paper specifically to maintain a higher degree of native fibrinogen in the assay.

### 3.2 Thrombolysis and fluorometric monitoring of whole blood clot fibrinolysis

Thrombolysis, in particular fibrinolysis, was accomplished utilizing tPa introduced into human pooled plasma. Thus, leading to activation of both plasminogen within the plasma itself, in addition to plasminogen incorporated within the clot analogs through the process of clot formation. Importantly, as platelet contraction affects clot permeability that can cause a significant reduction in fibrinolysis, whole blood clot analogs utilized in this study were formed in the Chandler loop for 1 h to allow for a complete platelet contraction to ensure consistent clot permeability despite clots being visually stable within 15 min after clot initiation.^34^ We aimed to understand fibrinolysis of whole blood clots via two primary means commonly presented in the literature—static and dynamic digestion, with dynamic further subdivided into Chandler loop versus RT‐FluFF using constant shear flows.

Inherit limits of fibrinolysis detection are reliant on the degree to which FITC‐Fg is released into solution and the extent to which fluorescence signal is quenched by other particles in the solution—including FITC‐Fg self‐quenching. Fibrinolysis was able to be detected at our lowest tPa concentration of 40 ng/mL in the RT‐FluFF assay, as seen by a rise in the rate of FITC‐Fg release compared to baseline; however, this difference was not statistically significant. We propose that the reason for the blunted rise in fluorescence signal stems from potential fluorescence quenching triggered by a faster release of loosely incorporated RBCs from the surface of the clot. The intensity of released FITC‐Fg was quenched as hemoglobin absorbs photons near the wavelength at which FITC emits them upon excitation.^35^, ^36^ We also documented inherit FITC‐Fg quenching in the experiments comparing rates of fluorescence rise between fluorescein and FITC‐Fg infusions. This, inherent FITC‐Fg quenching could also contribute to smaller rises in fluorescence than anticipated. The relatively small amounts of FITC‐Fg released at the 40 ng/mL of tPa condition are not able to significantly overcome the effects of RBC fluorescence quenching that stems from the initial burst release of RBCs loosely coating the clot surface. On the other hand, higher concentrations of tPa lead to large increases in FITC‐Fg release thus diminishing the impact of the RBCs. Additionally, the inherent rise of baseline plasma fluorescence over the course of 60 min of digestion in the RT‐FluFF assay also means a decrease in the absolute difference between our control and 40 ng/mL group, thus making statistical significance more difficult. As anticipated, greater concentrations of tPa led to more rapid rises in FITC‐Fg release, increased fibrinolysis, regardless of the digestion modality.

Although tPa concentrations were conserved between all digestion modalities, mechanical dissolution of the clots stemming from shear forces was not identical. It is these mechanical forces that drive clot mass loss as RBCs get released from the clot upon fibrinolysis.^37^ In the static digestion condition, minimal shear forces are present and thus RBC washout is minimal, leading to smaller degrees of clot mass loss compared to dynamic conditions. Both dynamic conditions utilizing pulmonary wall shear (400–600 s^−1^)—Chandler loop (464 s^−1^) and RT‐FluFF assays (398 s^−1^)—show a similar degree of clot mass loss over a large range of tPA concentrations. However, these results do not necessarily validate an interchangeable use of the two dynamic setups under other shear or clot digesting conditions in which plasma coagulation factors contribute to overall clot digestion dynamics where the larger system volume of the flow loop and reservoir provides added physiological relevance beyond the Chandler loop. This also calls for additional experiments to comprehensively compare RT‐FluFF with the Chandler loop for thrombolysis and drug profiling in the future. Furthermore, increased RBC release is also suggested by the fact that the RT‐FluFF assay does seem to maintain a slightly higher degree of percent clot mass lost at comparable tPa concentrations than those in the Chandler loop despite the latter setup having a slightly higher wall shear rate. This rise stems both from direct increased fibrinolysis and increased RBC release due to mechanical shear forces from pressure‐driven flow acting on a degrading fibrin network.^38^ Additional thrombolysis also stemmed from the mechanical effects of shear in the realm of facilitating dynamic plasminogen interactions and tPA permeations into the clot analogs themselves. Prior publications have shown that increasing levels of shear, as well as turbulent flow, allow for greater plasma/plasminogen penetration into the interior fibrin network of clots.^8^, ^39^

Correlation analyses conducted for each digestion modality indicated that static digestion protocols had the highest correlation of clot mass lost to rate of rise in FITC‐Fg concentrations. Meanwhile, both dynamic fibrinolysis experimental setups maintained lesser, yet similar, degrees of linear correlation between clot mass loss and a rise in fluorescence. These results are expected when bearing in mind the prior discussion of the importance of mechanical shear forces during thrombolysis. Overall, less linear correlation was seen because a given rise in FITC‐Fg release is met by a greater reduction in clot mass than expected based on static digestion, attributable to greater non‐fibrinolytic clot mass loss from RBC extrication secondary to mechanical forces induced by shear.^40^, ^41^ Additionally, there is increased potential for FITC‐Fg to adhere to the tubing within the Chandler loop or RT‐FluFF assay compared to simple static digestions, thereby also decreasing the linear correlation coefficients.

### 3.3 Shear and pulsatile impact on plasma clot fibrinolysis

Higher shear rate could contribute to drug replenishment, drug permeation, and increased mechanical clot tearing. Studies have shown increased drug‐induced fibrin clot dissolution in parallel plate setups at higher shear rate.^42^, ^43^ Results from the RT‐FluFF assay are in accordance with these findings where significantly faster tPA‐induced plasma clot dissolution is observed in most groups at high shear (913 s^−1^) compared to those at lower shear (205 s^−1^).

The effect of pulsatile flow on thrombolytic drug activities has been poorly addressed in the literature. Pulsatile pressure amplitude can have a huge variation due to diversified vessel geometry and pathological conditions in human vasculature. For instance, it can be 25 mmHg in the right ventricle and 15 mmHg in the pulmonary artery of a normal patient and as high as 80 mmHg in pulmonary artery hypertension patients.^44^, ^45^ Our results have suggested an increasing trend of drug‐induced clot digestion rate at increased pulsatile pressure amplitude (4–40 mmHg) or decreased dampener size (60–6 cc). Statistical significance was observed between 4 and 40 mmHg at 913 s^−1^ for a 19% increase and between the control and additional heartbeat (1 Hz) pulsation at 523 s^−1^ for a 63% increase. These results indicate a higher impact of pulsatile pressure amplitude on clot dissolution at higher average wall shear conditions, which could potentially help design better thrombolytic therapy for patients with impaired hemodynamics in larger blood vessels, for example, PE patients with pulmonary hypertension. Apart from the comparison of experimental results under different pulsatile levels, this manuscript introduced a method for creating mimetic pulsatile flow using a peristaltic pump. Through matching dimensionless factors, including length scale, *Re*, and Womersley numbers of a human MPA, one could establish human MPA‐relevant pulsatile flow dynamics in the unbranched tubing. Importantly, the model version described herein is a simplified demonstration as it focused on using the same averaged *Re* over a cardiac cycle; however, efforts can be spent to further match transient *Re* values at systolic and diastolic phases of the cardiac cycle to dictate a much closer kinematic similarity of MPA pulsatile flow. In the MPA‐relevant model condition (averaged wall shear = 913 s^−1^), the 20 cc dampener giving a pressure amplitude of 20 mmHg more closely captures the native MPA with a pressure difference of ~15 mmHg.^46^ We used fluorescently labeled plasma clots instead of whole blood clots for these tests to better understand the fundamental effect of pulsatility on fibrinolysis. Future works could implement whole blood clot tests at these pulsatile conditions to study the impact of pulsatility on thrombolysis.

### 3.4 Outlooks and limitations of the RT‐FluFF assay

Given the distinct clot components and distributions in arterial and venous thrombi, platelet‐rich arterial and RBC‐rich venous whole blood clot analogs could be formed by adjusting shear conditions in the Chandler loop and placed in the RT‐FluFF setup to facilitate more representative thrombolysis and drug testing tailored to a specific in‐vivo clot target. Clot contraction and aging could be adjusted by using different clot initiation methods, modulating clot formation time, or spiking inhibitors or agonists during clot formation in the Chandler loop. The RT‐FluFF setup also makes it feasible to study bolus and continuous infusion and synergistic effects of anticoagulant and thrombolytic drugs in a macroscopic manner. The pump rate and reservoir elevation of the RT‐FluFF setup could also be easily adjusted to provide for desired wall shear stress and flow pressure to mimic various hemodynamics in human blood vessels. Portions of the tubing could also be replaced by a 3D‐printed branched vessel geometry for more complex and physiologically relevant digestion studies incorporating diverse vessel structures.

One limitation of the RT‐FluFF assay remains to be the in‐vitro nature of the setup as it cannot offer information on drug toxicity and pharmacokinetics despite perfusion of human blood or plasma. The recent advancements of intravital microscopy make it possible for real‐time imaging of thrombolysis in animals.^47^, ^48^ Since animals, especially small rodents, bear distinct physiology and hemodynamics to that of humans, the RT‐FluFF assay offers complementary drug testing results in the preclinical phase in a more human physiologically similar system without requiring live animal experimentation. Although Chandler loop‐formed clots bear good resemblance to native thrombi, the flow conditions generated in the device do not perfectly reflect physiologic flow conditions. The circular geometry is prone to the formation of Dean flow.^49^ This could mean clots formed in the device are a less direct reflection of the effects of shear. Thus, the present in‐vitro platform can genuinely benefit from the utilization of clot analog formation techniques ideally incorporating pulsatile flow, or even utilizing retrieved patient thrombi labeled with FITC‐linked fibrin binding peptides.^50^ The portable fluorometer we designed is capable of tracking FITC fluorescence signals in plasma, as plasma has low background fluorescence values with the wavelengths used. However, fluorescence signals can be significantly quenched by RBC release even at levels as low as 0.1% v/v within the system. This is in part due to a 550 nm sensitive photo sensor used for signal acquisition, a wavelength overlapping with RBC absorption at 538 nm.^36^ Although experiments were not largely affected, since our digestion medium was plasma, different fluorophores or different photosensors would be recommended for studies that aim to utilize whole blood as a medium. Additionally, reliance on FITC‐fibrinogen presents the issue of fluorescence quenching due to FITC residue burial within the protein complex.^51^ To ensure stable shear with laminar flow was achieved in the RT‐FluFF assay, a dampener was utilized to reduce pressure fluctuations associated with the peristatic pump. While this provides a high degree of digestion control and consistent flow field development, it does not fully encompass physiologic pulsatile flow.

## 4 CONCLUSIONS

Thrombolysis and fibrinolysis are very dynamic processes whose accurate characterization requires numerous repeat observations over a short period of time. Thus, commonly utilized means of quantifying fibrinolysis/thrombolysis such as endpoint reads, whether changes in mass or absolute rises in fluorophore release, may not truly capture the entire dynamic process. The RT‐FluFF model can help bridge this gap by providing a means of real‐time fluorescence monitoring in the context of a flowing system with high fidelity. This allows users to capture both the instantaneous rates of fibrinolysis in addition to dynamics such as initial lag phases, representing both tPa binding and infiltration, as well as mechanical clot digestion effects. Varying pulsatile pressure amplitude can affect tPA efficacy on clot digestion. Increasing pressure amplitude from 4 to 40 mmHg results in an increased trend of tPA‐induced clot digestion at a variety of pulsatile flow conditions including human MPA‐mimicked pulsatile flow (average wall shear rate = 913 s^−1^) and slower pulsatile flows (205 and 523 s^−1^). A human heartbeat mimetic setup further confirmed the impact of pulsatile difference on tPA‐induced thrombolysis. Knowing the difference in tPA‐induced thrombolysis at varying pulsatile pressure difference can help understand the mechanism of thrombolytic therapy in native vessels. The present findings also suggest that incorporating a pulsatile flow in an in‐vitro model is necessary to provide a dynamic and potentially more representative platform for evaluating thrombolytic drugs.

In addition to mimicking in‐vivo flow, and allowing for live fluorescence monitoring, our system also maintains numerous other benefits: (a) interchangeable tubing geometries/configurations, (b) real‐time pressure monitoring as a means of quantifying degree of lumen occlusion, (c) real‐time fluorescence measurements requiring no system intervention, (d) reading duration/frequency flexibility, (e) incorporation of a reservoir to better recapitulate in‐vivo conditions, (f) flow‐patterns generated resemble in‐vivo conditions since the substrate is stationary with fluid flow‐by, in contrast to the Chandler loop, (g) option to include pulsatile flow, and (h) live‐imaging of digesting clot substrate allowing for image analysis. Overall, the RT‐FluFF system serves as a cost‐effective and versatile in‐vitro testing platform ideal for use in the development and screening of novel thrombolytic agents.

## 5 METHODS

### 5.1 Ethics

Informed consent was obtained from all subjects, and/or their legal guardians, before blood donation. All experimental protocols were approved by the IRB ethics committee at Indiana University School of Medicine, USA.

### 5.2 Whole blood and plasma processing

Venous whole blood was collected from healthy volunteers (*n* = 5) by a phlebotomist in accordance with guidelines/methods outlined in our institutionally approved IRB protocol (1610652271). All collection and handling of human specimens have been priorly approved by the IRB at our institution. Blood was collected into 3.2% Sodium‐Citrate tubes and immediately pooled into 15 mL tubes for use (BD Vacutainer). Sample hematocrits were calculated utilizing microhematocrit tubes centrifuged at 2750*g* for 3 min (Thermo Fisher Scientific). Whole blood was stored at 4°C and brought to room temperature (RT) for over 30 min before use. Human plasma units were donated on behalf of the Eskenazi Blood Bank for research use and were aliquoted before storage at −20°C.

### 5.3 FITC‐fibrinogen synthesis

Fibrinogen was labeled with FITC as previously described.^21^ In short, lyophilized human fibrinogen and FITC (Sigma‐Aldrich) were reconstituted in a phosphate‐buffered saline (PBS)—10% dimethyl sulfoxide (DMSO) solution. 200‐fold excess FITC was reacted with fibrinogen at RT for 18 h to attain 14‐FITC per fibrinogen molecule (<8% DMSO in reaction solution). Tagged fibrinogen was purified using 100 kDa cutoff filters following manufacturer recommendations (Amicon®, Millipore). The average amount of FITC per fibrinogen was verified.^21^ FITC‐Fg aliquots were then made to a final concentration of 3 mg/mL and stored at −20°C.

### 5.4 Thromboelastography

TEG was utilized to capture baseline clotting profiles. TEG was run in accordance with manufacturer‐provided protocols and priorly‐published protocols utilizing Haemonetics TEG‐5000 instruments (Haemonetics, Boston, MA).^52^ In brief, 1 mL of citrated whole blood was mixed into 40 μL of kaolin and followed by the introduction of 340 μL of the mixed sample into non‐coated TEG cups containing 20 μL of 12 mM CaCl_2_. Clotting was monitored at 37°C. Incorporation of FITC‐Fg into our samples for TEG analysis was performed before introducing the whole blood into the kaolin. Ratios of FITC‐Fg utilized were 50:1, 10:1, and 5:1, which corresponds to 0.18, 0.88, and 1.76 μM, respectively. A vehicle control that contained PBS was also included in analysis to account for potential dilution of the whole blood samples upon addition of the FITC‐Fg. Samples were run in triplicate.

### 5.5 Whole blood clot formation under shear

Whole blood clots were formed utilizing a Chandler loop to mimic exposure to physiologic levels of shear. In brief, FITC‐Fg was added to citrated whole blood at a final ratio of 10:1 (0.88 μM) before recalcification. After gentle inversion of the mixture, the whole blood was recalcified utilizing CaCl_2_ to achieve a final concentration of 11.8 mM. Recalcified whole blood was loaded, using a syringe, into 5/32 in. tubing (Tygon 100‐65 medical tubing) such that half of the volume of the end‐joined loop would remain empty—final volume of whole blood utilized ~2 mL (U.S. Plastics). The tubing loops were immediately placed on a rotating semi‐submerged drum of 5.5 cm radius to begin clot formation. The drum was set to rotate at 40 RPM (calculated shear: 464 s^−1^) for 60 min. Partial submersion of the Chandler Loop drum in a water bath ensured clot formation was conducted at a constant 37°C. Upon completion, individual clots were weighed and imaged under both white light and UV light. Clots were subsequently re‐submerged in their residual serum to ensure they remained hydrated during waiting periods.

### 5.6 H&E staining and epifluorescence

A single representative clot formed in the Chandler loop was collected from each subject and stored in 10% neutral buffered formalin for 24 h before being placed in 70% EtOH. Once preserved, clots were submitted to the Indiana University Histology Core for sectioning and staining with H&E. Analysis of H&E slides was performed utilizing threshold analysis in ImageJ to isolate contributions of RBCs, white blood cells, and fibrin as part of clot composition. Non‐stained sections from the same clots were utilized for epifluorescence imaging utilizing an LSM 800 confocal microscope (Zeiss).

### 5.7 Static digestion protocol

A subset of the FITC‐Fg‐labeled whole blood clots previously described was subjected to fibrinolysis under static conditions. Frozen plasma was thawed at 37°C and aliquoted to subsequently be loaded with Alteplase at concentrations of 0 (i.e., no Alteplase added), 40, 200, or 1000 ng/mL (Genentech). Clots were gently loaded into 1.5 mL centrifuge tubes and filled to the 1.5 mL mark with plasma. Subsequently, tubes were submerged in a 37°C water bath for the duration of the 60‐min digestion. Triplicate samples of the plasma were taken at 0‐, 30‐, and 60‐min marks of digestion for fluorescence quantification. A spectrophotometer (Molecular Devices, SpectraMax M5) was utilized for all readings. Settings for the spectrophotometer were as follows: 495 nm excitation, 519 nm emission, and 515 nm auto‐filter. Clot weights were also recorded at the end of the digestion period to calculate the percent clot mass lost.

### 5.8 Chandler loop digestion protocol

A second modality of digestion explored was utilizing the Chandler loop configuration to also conduct fibrinolysis under dynamic conditions. Frozen plasma was thawed at 37°C and aliquoted to subsequently be loaded with Alteplase at concentrations of 0 (i.e., no Alteplase added), 40, 200, or 1000 ng/mL. FITC‐Fg‐labeled whole blood clots were submerged in weigh‐boats containing 5 mL of plasma with varying tPa concentrations. A syringe attached to a new 5/32 in. Tygon tube was used to draw in both the plasma and clot into the tubing (U.S. Plastics). A final volume, including the clot, of 3 mL was drawn into the tubes each time. End‐joined tubes were placed onto the Chandler Loop and rotated at 40 RPM (464 s^−1^) for the duration of 60 min. Plasma was sampled at 0, 30, and 60 min and read via spectrophotometer. Clot masses were once again collected at the end.

### 5.9 RT‐FluFF assay construction

A physiological scale in‐vitro blood flow device was developed to offer laminar flow at an adjustable shear rate for clot analog digestion experiments. The flow device consists of a peristaltic pump system—Masterflex EasyLoad II pump and 1.5 ft #15 peristaltic tubing (Masterflex), a 50‐mL tube reservoir (Corning Scientific), and a flow dampener, which are connected by 5/32 in. Tygon tubing pieces. To offer a non‐contact flow at a desired output, a peristaltic pump was used. The dampener further mitigates the peristalsis by the pump to provide an approximate laminar flow pattern. Dampeners were made via fitting a 5/16 in. (inner diameter = 0.008 m) tubing segment to the tip of three different‐sized plastic syringes (6, 20, and 60 mL) noted as 6, 20, and 60 cc. A three‐way connector was used to incorporate the dampener into the flow loop. Plunger positions of dampeners were adjusted to be spontaneously balanced at a 187 RPM pump rate. This resulted in 1, 5, and 60 mL in 6, 20, and 60 mL syringes yielding pressure differences between systolic and diastolic of 4, 20, and 40 mmHg, respectively. Two disposable pressure sensors (sensors A and B) and a monitor (Siemens SC 7000) were employed to provide pressure‐drop reads between two positions to confirm the model abided by the Navier Stoke equation. The real‐time clot digestion monitoring was achieved by incorporating a portable fluorometer positioned directly after the clot. The fluorometer includes a flow chamber cuvette, 450 nm laser diode, 520 nm long pass filter, and a 550 nm sensitive photodetector. Signal was processed by an operational amplifier circuit forwarding to an Arduino micro‐chip for recording (Arduino). Fluorescence reads were obtained every 30 s by default unless otherwise specified. To guarantee a fully developed laminar flow pattern, clot analogs were positioned at a calculated entrance length from the last bifurcation unless otherwise specified.

### 5.10 MPA mimetic pulsatile flow setup

The system was adapted to create an in‐vitro MPA flow condition to explore the impact of various pulsatility levels (4, 20, and 40 mmHg or ±2, 10, and 20 mmHg) on thrombolytic drug‐induced clot digestion.^22^ Given that average blood output in MPA is about 5.2 ± 1.0 L/min where average *Re* = 1570 ± 404 with a maximum volumetric flow of 21 L/min, the in‐vitro flow model should be down‐scaled to a level that maintains the system fidelity and saves experimental material.^53^ To mimic the kinematic dynamics in MPA, parameters in the RT‐FluFF model were adjusted to match an averaged *Re* where $\bar{Q}$ is the averaged volumetric flow rate of a pulsatile cycle, D is the lumen diameter, and ν is the kinematic viscosity using the following equation:

$$\frac{{\bar{Q}}_{\text{model}}}{D_{\text{model}}\nu_{\text{model}}}=\frac{{\bar{Q}}_{\mathrm{MPA}}}{D_{\mathrm{MPA}}\nu_{\mathrm{MPA}}}$$

The fanning friction factor is dependent on *Re*. The Womersley number is also matched using the equation below where f is the pulsatile frequency:

$$D_{\text{model}}\sqrt{\frac{f_{\text{model}}}{\nu_{\text{model}}}}=D_{\mathrm{MPA}}\sqrt{\frac{f_{\mathrm{MPA}}}{\nu_{\mathrm{MPA}}}}$$

Additionally, the diameter of the peristaltic in‐pump tubing, which is different from the $D_{\text{model}}$, should be selected based on the calculated volumetric flow output ${\bar{Q}}_{\text{model}}$ of the peristaltic pump. Since model pulsatile frequency $f_{\text{model}}$ is dictated by the product of roller numbers and revolution per minute of the pump, which also dictates ${\bar{Q}}_{\text{model}}$, the following equation should also be obeyed.

$${\bar{Q}}_{\text{model}}=1\;L/\min*\frac{f_{\text{model}}}{40}$$

Flow parameters in the in‐vitro model can be solved using the three equations. Clinical MPA data were used as a guide while actual Re and alpha values were calculated based on the basic properties shown in Table S1.^54^ The reservoir is elevated to match average pressure at pressure sensor B (the second pressure sensor) to an average human MPA pressure which is 12 mmHg. A fluorescently labeled clot substrate is fixed immediately before pressure sensor B to match the ratio of length scales of the MPA. The calculated model condition provides an MPA‐relevant flow dynamics to study the effect of pulsatile pressure amplitude on thrombolysis. By adjusting the dampening efficiency to the oscillation generated by the peristaltic pump, pulsatile pressure amplitude can be further tuned in the RT‐FluFF model, where the flow can have different transient *Re* although an average value is still maintained. In all experiments, liquid only oscillates in the fitted tubing segment without going into the syringe indicating a good retaining of total circulating fluid. The human heartbeat setup, noted as 1 Hz on/off, was achieved by modifying peristaltic pump to include an in‐house developed finger‐bot that pushes the pump on/off button to give a pulsatile flow frequency of 1 Hz. Flow turned on and off at a relatively instant manner generating an approximately sinusoidal wave. The waveform can be confirmed by both pressure sensors A and B in the RT‐FluFF. At the largely dampened (4 mmHg, 60 cc) 214 RPM pump condition, the pressure curve revealed a smooth oscillation and gave a volumetric output at the same level as the 107 RPM (average wall shear rate = 523 s^−1^) pump flow (Figure S10).

### 5.11 RT‐FluFF assay digestion protocol

FITC‐Fg‐labeled whole blood clots were employed in the RT‐FluFF assay for digestion in the constant shear setup. FITC‐Fg‐labeled platelet‐free plasma clots were employed for all pulsatile flow tests. Each clot was fixed in the tubing using a 31‐gauge syringe needle at one‐tenth the distance from the clot head. To mimic human pulmonary flow conditions, the reservoir was heated at 37°C and lifted to 8 cm above the clot level to give an average flow pressure of 12 mmHg, and the pump rate was either adjusted to generate a 398 s^−1^ wall shear flow for the constant shear setup or multiple other wall shear flows at different pressure amplitudes. For each experiment, the model was perfused with newly thawed pooled plasma with premixed Alteplase. Clot digestion was monitored utilizing the in‐line fluorometer. Flow pressure, clot appearance, and clot break‐off times were recorded.

### 5.12 Shear‐stretch analysis

Chandler loop‐formed whole blood clots were fixed in the RT‐FluFF assay tubing using a 31‐gauge syringe needle at one‐tenth the distance from the clot head. PBS flowed through the system at various rates of shear including 0, 300, 600, and 900 s^−1^. Clots were allowed to stretch and equilibrate at each shear for 2 min before video capture. Quantification of the video frames was conducted utilizing ImageJ in which clot length could be accurately measured from clot head to clot tail. Percent change in length could then be calculated based on respective initial lengths.

### 5.13 Data analysis

All data were collected/processed using Microsoft Excel. ANOVA analysis was used to ascertain statistical differences among groups with three or more conditions, followed by a Tukey test for individual subset comparisons. Student *t* tests were utilized to compare two categorical variables with each other. Statistical significance was deemed to be a *p* value < 0.05.

## AUTHOR CONTRIBUTIONS

**Ziqian Zeng:** Conceptualization (equal); data curation (equal); formal analysis (equal); writing – original draft (equal); writing – review and editing (equal). **Alexei Christodoulides:** Conceptualization (equal); data curation (equal); formal analysis (equal); writing – original draft (equal); writing – review and editing (equal). **Nathan J. Alves:** Conceptualization (lead); data curation (equal); resources (lead); writing – review and editing (equal).

## CONFLICT OF INTEREST STATEMENT

The authors declare no conflict of interest.

## Supporting information


**DATA S1.** Supporting Information

## Data Availability

Data can be made available upon request from the corresponding author.
