# Supplementary material for: Real‐time tracking of fibrinolysis under constant wall shear and various pulsatile flows in an in‐vitro thrombolysis model
Source: Bioeng Transl Med. 2023 Apr 11;8(3):e10511. doi: 10.1002/btm2.10511 (PMC10189439; doi:10.1002/btm2.10511)
Supplement: Supplementary file 1 — DATA S1. Supporting Information [file BTM2-8-e10511-s001.docx]

**Real-Time Tracking of Fibrinolysis Under Constant Wall Shear and Various Pulsatile Flows in an *in-vitro* Thrombolysis Model**Ziqian Zeng ^a,b, †^, Alexei Christodoulides ^a, †^, Nathan J. Alves ^a,b^
^a^ Department of Emergency Medicine, Indiana University School of Medicine, Indianapolis, IN, USA
^b^ Weldon School of Biomedical Engineering, Purdue University, West Lafayette, IN, USA

^†^*Both authors contributed equally to this work*

*Corresponding Author* – Dr. Nathan J. Alves – [nalves@iu.edu](mailto:nalves@iu.edu)
*Department*: Department of Emergency Medicine, Indiana University School of Medicine, Indianapolis, IN, USA
*Address*: 635 Barnhill Dr. MS2063, Indianapolis, IN 46202

**Supplemental Figures**

Table of Contents:

Supplemental Table 1 – Flow Parameters of RT-FluFF model

Supplemental Figure 1 – Thromboelastography Clotting Parameters

Supplemental Figure 2 – Varying FITC-Fg Ratios: Chandler Loop Clot Digestion

Supplemental Figure 3 – RT-FluFF Device Picture

Supplemental Figure 4 – RT-FluFF Dampening

Supplemental Figure 5 – Absolute System Pressures

Supplemental Figure 6 – Shear-Induced Clot Stretching

Supplemental Figure 7 – Individual Patient Fluorometer Scatter Plots

Supplemental Figure 8 – Rates of FITC-Fg Release from Chandler Loop and Static Digestions

Supplemental Figure 9 – Clot Mass Loss% Compared across Various Pulsatile Conditions

Supplemental Figure 10 – Pressure Waveform for 1Hz on/off Heartbeat Flow

**Supplemental Table 1.** Flow Parameters of RT-FluFF model to mimic MPA

|  | **Human MPA** | **RT-FluFF Model** |
| --- | --- | --- |
| Viscosity (pa·s) | 0.0035 | 0.0012 |
| Density (kg/m^3^) | 1060 | 1025 |
| Frequency (Hz) | 1 | 16.76 |
| Lumen diameter (m) | 0.025 | 0.004 |
| Volumetric flow rate (L/min) | 5.2 | 0.268 |
| **Reynolds number** | 1337 | 1337 |
| **Womersley number** | 17.2 | 17.2 |

Note: Human MPA, blood and plasma data are collected from a clinical study. (Sloth et al. 1994)

**Supplemental Figure 1**

*****

******

*******

*****

*Supplemental Figure 1:* Bar plots outlining the TEG parameters MA, TMA, and angle in the various FITC-Fg groups explored during optimization. Single asterisk denotes P-value < 0.05, Double asterisk denotes P-value <0.01, Triple asterisk denotes P-value <0.001. Note the effect on clotting parameters by the 5:1 ratio, with minimal perturbation seen in the 10:1 groups.

**Supplemental Figure 2**

*Supplemental Figure 2:* Bar plot outlining percent clot mass lost during digestion of Chandler-loop made clots utilizing various ratios of FITC-Fg. Clot digestion conducted over the course of 60-minutes with 600 ng/mL tPa. Trend to suggest perhaps increased clot dissolution with increasing FITC-Fg incorporation, however, no statistical significance is seen.

**
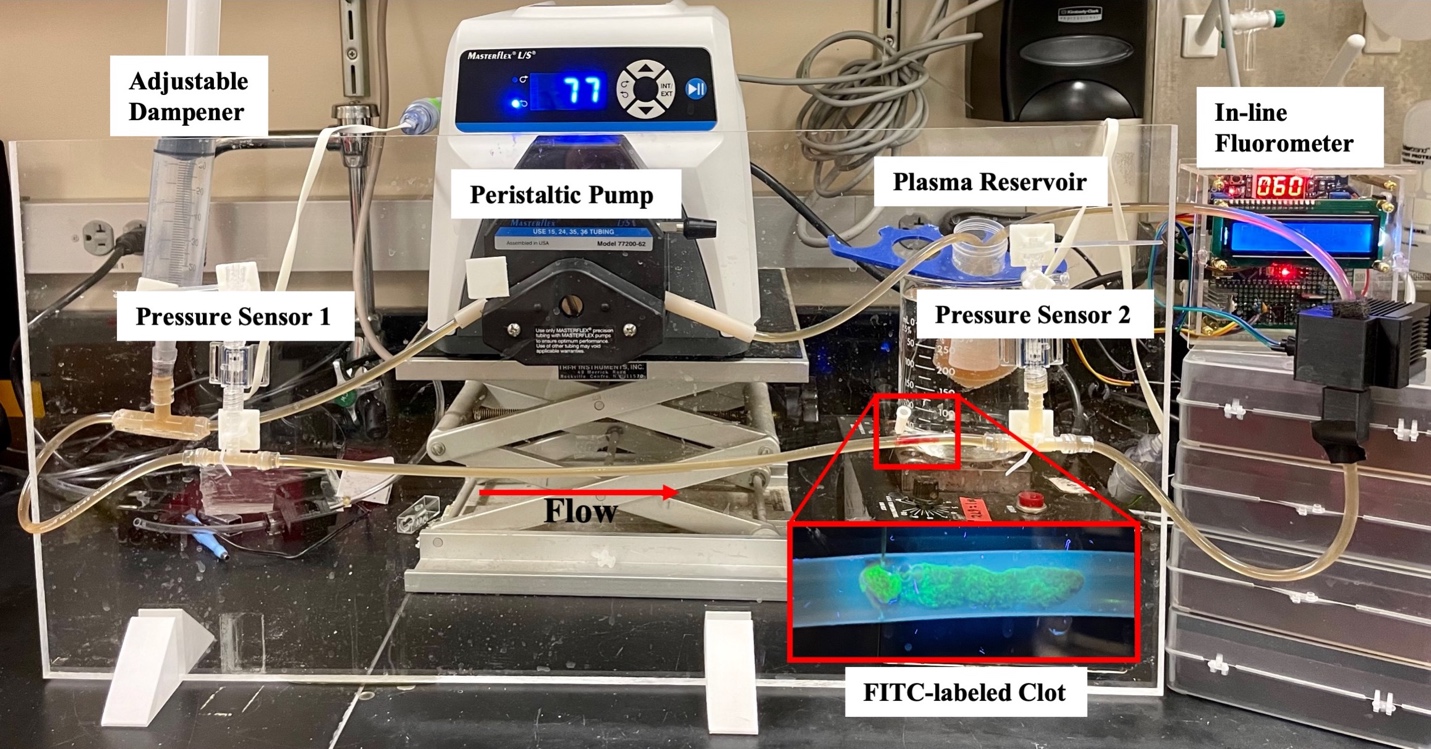
Supplemental Figure 3**

**Supplemental Figure 4:**

*Supplemental Figure 4:* DeltaP (Systolic Pressure – Diastolic Pressure) outlined in pressure sensor 1 and 2 in various systemic pressure settings as well as in dampened versus undampened settings. Height measurements refer to the height of the output drain relative to the pressure sensors as this was adjusted to raise/lower systemic pressures without the need to change pump RPM for a given desired level of shear. Note that in the range of shears we utilized for our experiments, we were in the range where dampening was maximally effective.

**Supplemental Figure 5:**

*Supplemental Figure 5:* Absolute systolic pressure readings at pressure sensor 1 and 2 over a wide range of pump shear rates.

**Supplemental Figure 6:**

*Supplemental Figure 6:* Shear-induced clot analog stretching. Individual colored bars represent respective levels of shear (s^-1^) from the peristaltic pump. Change in length (%) is relative to the clot length under stagnant conditions, i.e., 0 s^-1^ of shear. Note the relative uniform stretching of clots in response to varying levels of shear.

**Supplemental Figure 7:**

*Supplemental Figure 7:* Scatter plots from remainder four patients. Each point represents a single reading from the fluorometer part of the RT-FluFF assay. Rates of fibrinolysis were derived from the slopes of the linear regions in each graph. Of note, certain groups had a shortened linear region resulting from a prolonged lag phase; however, rates of FITC-Fg release were still able to be extracted from shortened linear phases.

**Supplemental Figure 8**

******

*******

*******

******

*******

*******

*Supplemental Figure 8:* Rates of FITC-Fg release at various tPa concentrations. Top figure pertains to the static digestion protocol and the bottom figure is digestion seen in the Chandler loop. Note the rise in rate of FITC-Fg release as tPa concentrations are increased in either digestion modality.

**Supplemental Figure 9**


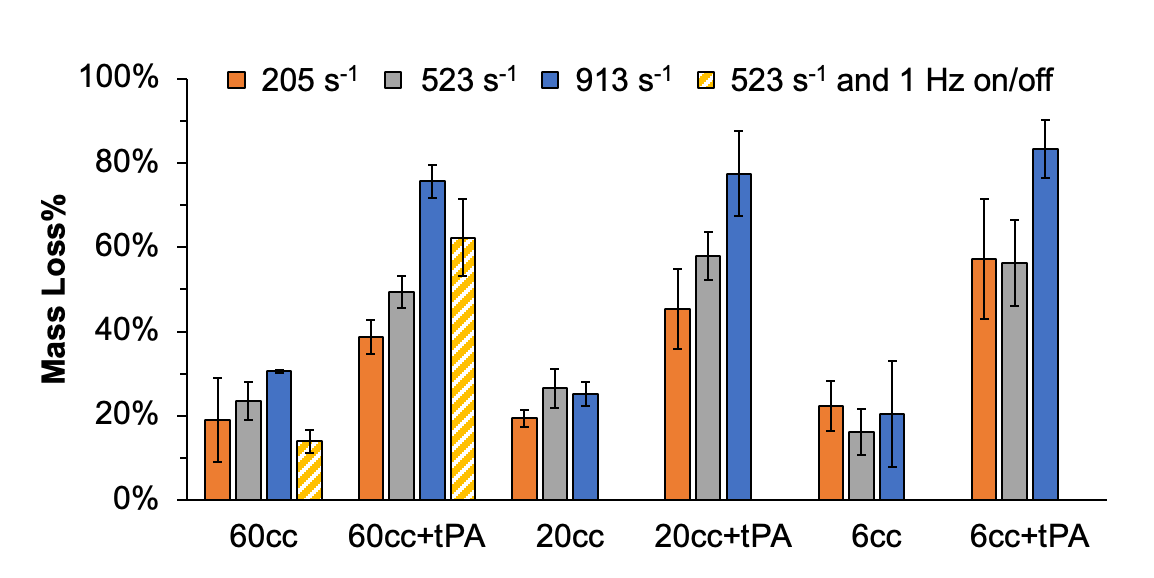


*Supplemental Figure 9:* Clot mass loss% of fluorescently labeled plasma clots at 30 min were compared across three dampeners (60, 20, and 6 cc) at averaged shear rates of 913 s^-1^, 523 s^-1^, 205 s^-1^, and 523 s^-1^ at 1 Hz on/off.

**Supplemental Figure 10**


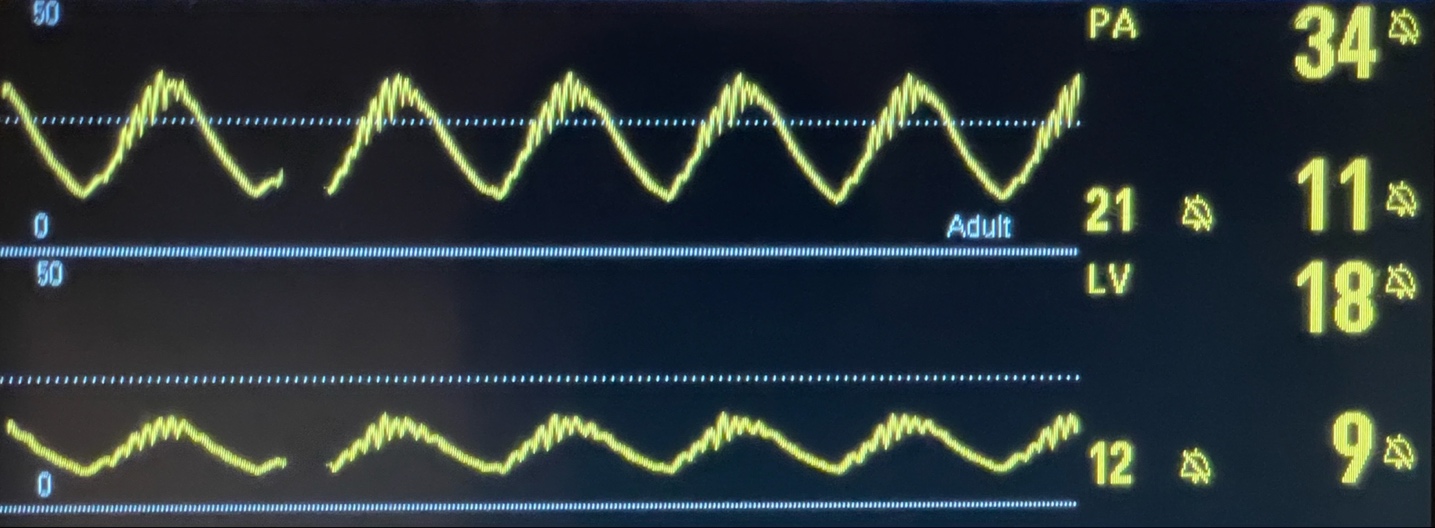


*Supplemental Figure 10:* The pressure waveform monitored for the 1Hz on/off heartbeat setup by pressure sensor B via Siemens SC 7000. A 60 cc pulsatile flow dampener was utilized to first provide for a maximally dampened flow. The heartbeat setup was then achieved by using a fingerbot pushing the on/off button to generate an oscillation at 1 Hz.
